# Selective fluorescent probe for Tl^3+^ ions through metal-induced hydrolysis and its application for direct assay of artificial urine†

**DOI:** 10.1039/d4ra06726f

**Published:** 2025-01-15

**Authors:** Myung Gil Choi, Yerin Jang, Mi Gang Kim, Sangdoo Ahn, Suk-Kyu Chang

**Affiliations:** a Department of Chemistry, Chung-Ang University Seoul 06974 Republic of Korea sangdoo@cau.ac.kr skchang@cau.ac.kr +82 2 825 4736 +82 2 820 5230

## Abstract

In this research, we report a simple fluorescent probe designed to detect thallium(iii) ions (Tl^3+^) in artificial urine samples. The Tl^3+^ signaling probe (TP-1) was readily prepared from 2-acetyl-6-methoxynaphthalene and hydrazine. In a pH 4.8 acetate buffer solution containing 1% (v/v) *N*,*N*-dimethylformamide as a solubilizer, probe TP-1 exhibited turn-on fluorescence signaling behavior in the presence of Tl^3+^. Other metal ions, anions, and major urine components such as uric acid, urea, and creatinine did not produce any noticeable fluorescence changes. The Tl^3+^ signaling of TP-1 was attributed to the hydrolysis of the hydrazone moiety, yielding the parent fluorophore 2-acetyl-6-methoxynaphthalene. The detection limit of TP-1 for Tl^3+^ sensing was 19 nM, and the signaling was completed within 2 min. Additionally, to further optimize the Tl^3+^ signaling of the hydrazone derivatives, we compared the effect of structural variations between the closely related ketone-hydrazone (TP-1) and aldehyde-hydrazone (TP-2) derivatives. We confirmed that the ketone-hydrazone (TP-1) demonstrated rapid and stable Tl^3+^ signaling behavior with satisfactory stability under the measurement conditions. Finally, as a practical application, a Tl^3+^ assay in artificial urine samples was performed using a smartphone as a portable signaling measurement and data analysis device.

## Abstract 2

A fluorescent signaling probe was developed for Tl^3+^ assay in artificial urine samples, demonstrating rapid response, high selectivity, and smartphone-based analysis.

## Graphical abstract

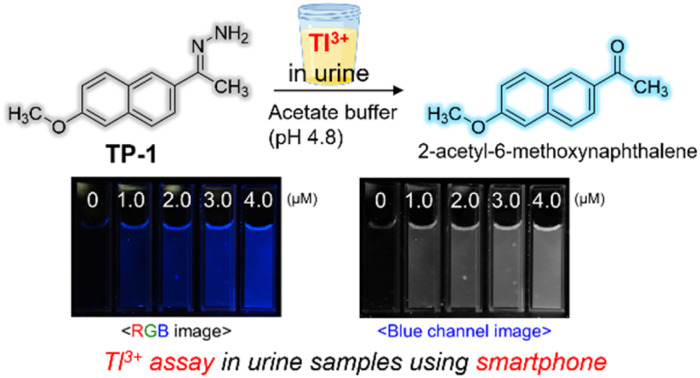

## 1. Introduction

Thallium, a Group 13 (boron group) post-transition metal, is well-known for its notorious toxicity in various environments.^1^ Despite its dangerous nature, thallium is used in several modern industrial fields, including the electronics industry for photoelectric cells and infrared detectors, and the manufacturing of glass with a high refractive index and low melting point.^2^ Additionally, thallium serves as an agent in nuclear medical scans and is utilized in medical products due to its resistance to acid and corrosion, as well as its antifriction properties.^3^ However, the widespread presence of thallium in the environment poses significant health risks,^4^ even at low concentrations, and causes severe neurological and gastrointestinal disorders.^5^ For these reasons, the United States Environmental Protection Agency (EPA) classifies thallium as a significant pollutant.^6^ Consequently, there is ongoing research interest in developing convenient and sensitive analytical methods specifically for detecting thallium.

Thallium primarily exists in two oxidation states: thallous (Tl^+^) and thallic (Tl^3+^) ions. It is known to be more toxic than other representative heavy metal ions, such as cadmium and mercury.^7^ Thallium(i) is more stable and prevalent than thallium(iii), but the latter is four times more toxic to humans and animals than the former.^8^ Traditionally, the determination of thallium has relied on standard instrument-based methods such as atomic absorption spectroscopy,^9,10^ inductively coupled plasma mass spectrometry,^11,12^ voltammetry/potentiometry,^13^ spectrophotometry,^14,15^ and fluorimetry,^16^ all of which are known for their high sensitivity and accuracy. However, these techniques require sophisticated instrumentation and are often costly, making them unsuitable for on-site, real-time monitoring of thallium levels. This limitation is particularly challenging in settings where rapid, accessible detection is crucial, such as in field-based or point-of-care scenarios. Therefore, the development of convenient and practical detection methods for thallium ions without resorting to complicated heavy instruments is necessary.

Various sensors and reaction-based molecular probes utilizing colorimetric and fluorometric responses have been actively investigated for the easy determination of toxic metal ions due to their selective complex formation ability and specific reactivity toward target species.^17,18^ These sensors and probes offer several advantages, such as simplicity, cost-effectiveness, portability, and the potential for real-time detection. Several thallium signaling sensors have been reported, employing the formation of selective host-guest type thallium complexes with hydroxamic acid and bis-pyridine ligands of the host system.^14,19^ Among the various exceptional thallium sensing approaches, reaction-based probes have recently emerged as a particularly attractive option due to their high sensitivity, remarkable selectivity, and rapid response times.^20^ For instance, the oxidation of trifluoperazine and arsenoxylphenylazo rhodanine derivatives has been investigated for the determination of Tl^3+^ ions in alloys, minerals, and urine samples.^21,22^ The oxidative coupling reaction between 3-methyl-2-benzothiazolinone hydrazone (MBTH) and 10,11-dihydro-5*H*-dibenzo[*b*,*f*]azepine (IDB) has also been reported for Tl^3+^ sensing in practical water samples and urine.^23^ Furthermore, oxidative hydrolysis of rhodamine sulfonhydrazide and hydroxamic acid has been identified as effective means for the colorimetric and fluorescent signaling for Tl^3+^ ions.^24,25^ The properties, signaling mechanisms, and practical applications of these reported Tl^3+^ signaling systems are summarized in Table S1 (ESI).†

A thallium-selective probe was designed by using well-established 2-acetyl-6-methoxynaphthalene as a signaling chromo-fluorophore and a hydrazone moiety as a signaling trigger. Hydrazones are highly useful and versatile compounds in organic and medicinal chemistry,^26,27^ and they are extensively utilized in the construction of metal–organic frameworks (MOFs), covalent organic frameworks (COFs), dynamic combinatorial chemistry, and as hole-transporting materials.^28^ Notably, numerous chemical sensors and probes based on hydrazone-containing molecules have been developed for detecting and visualizing chemically and environmentally significant metal ions, anions, and biologically important species.^29^ Hydrazone-based chemosensors for the determination of cyanide,^30^ fluoride,^31^ and acetate ions^32^ have been exploited through selective addition of the analyte or deprotonation of the hydrazone subunit. Additionally, several sensors incorporating hydrazone function as a binding site have been created for metal ion sensing through metal-hydrazone complex formation.^33–35^ In parallel to these, a number of hydrazone-based, reaction-based probes have been investigated for the determination of metal ions such as Cu^2+^ and Hg^2+^,^36,37^ as well as common oxidants like hypochlorite^38^ and peroxynitrite.^39,40^

In this research, we aimed to develop an easy and rapid method for the convenient measurement of urinary thallium levels of suspected acute thallium poisoning in the field. Initial clinical tests for the screening of thallium poisoning include urine tests, blood tests, and electrolyte tests that can easily check the patient's metal pollution status. Among these, analyzing thallium ions using urine with spectroscopic methods is more rapid and convenient due to the ease of sample collection and preparation as well as the simplicity of analysis without using complex equipment. We report the results obtained for a simple fluorescent signaling probe exhibiting useful fluorescence signaling for Tl^3+^ ions *via* the metal-assisted oxidative hydrolysis of a hydrazone moiety. We comparatively investigated the Tl^3+^ signaling behavior using two similar-structured ketone-hydrazone (TP-1) and aldehyde-hydrazone (TP-2) candidates. The thallium signaling of probe TP-1 ensures the rapid and convenient detection of the thallium level, without interference from common metal ions, anions, and major components of urine solution. The unique design of TP-1 offers not only a high selectivity for Tl^3+^ ions, even amidst common components in artificial urine, but also a rapid fluorescence response and compatibility with smartphone-based analysis, making it particularly suitable for field-based diagnostics. The practical application of the developed probe was ascertained by the successful determination of Tl^3+^ levels in artificial urine samples using merely a smartphone as a signaling measurement and analysis device. This advancement could be transformative in clinical and environmental settings where rapid, accessible detection of toxic metals is essential for timely intervention, using artificial urine as a readily available analyte for clinical testing.

## 2. Experimental

### 2.1 Preparation of Tl^3+^ signaling probes (TP-1 and TP-2)

Hydrazones of 2-acetyl-6-methoxynaphthalene and 6-methoxy-2-naphthaldehyde were synthesized following a modified procedure from the literature.^37^ For TP-1, 2-acetyl-6-methoxynaphthalene (0.40 g, 2.0 mmol) was placed in a 50 mL round-bottom flask and dissolved in 10 mL of ethanol. To this solution, an excess amount of hydrazine monohydrate (0.46 mL, 10.0 mmol) was added dropwise. The reaction mixture was stirred at room temperature for 12 hours. The resulting precipitate was then filtered and washed three times with 5 mL portions of ethanol. The product was purified by recrystallization from ethanol, yielding TP-1 as a white-yellow powder (0.38 g, 88.8% yield). The purity of TP-1 was verified to be >99% by high performance liquid chromatography.

Similarly, to prepare TP-2, 6-methoxy-2-naphthaldehyde (0.37 g, 2.0 mmol) was added to a 50 mL round-bottom flask and dissolved in 10 mL of ethanol. Hydrazine monohydrate (0.46 mL, 10.0 mmol) was then slowly added to the solution. The reaction was allowed to proceed with stirring at room temperature for 12 hours. Afterward, the precipitate was collected by filtration, washed thoroughly with ethanol, and purified by recrystallization from ethanol, yielding TP-2 as a white-yellow powder (0.37 g, 92.5% yield). The purity of TP-2 was determined to be >99% by high performance liquid chromatography.

The spectral data for both compounds are as follows:

TP-1: ^1^H NMR (600 MHz, DMSO-*d*_6_) *δ* 7.93 (d, *J* = 1.8 Hz, 1H), 7.91 (dd, *J* = 8.6, 1.9 Hz, 1H), 7.82 (d, *J* = 8.9 Hz, 1H), 7.71 (d, *J* = 8.6 Hz, 1H), 7.27 (d, *J* = 2.6 Hz, 1H), 7.13 (dd, *J* = 8.9, 2.5 Hz, 1H), 6.38 (s, 2H), 3.86 (s, 3H), 2.11 (s, 3H); ^13^C NMR (150 MHz, DMSO-*d*_6_): δ157.65, 142.61, 135.54, 133.97, 130.04, 128.82, 126.75, 124.13, 123.72, 118.86, 106.39, 55.62, 11.59; HRMS (EI^+^, *m*/*z*): calcd for C_13_H_14_N_2_O^+^ [M]^+^: 214.1106, found 214.1106.

TP-2: ^1^H NMR (600 MHz, DMSO-*d*_6_) *δ* 7.82 (s, 1H), 7.79 (d, *J* = 8.9 Hz, 1H), 7.74 (br s, 3H), 7.28 (d, *J* = 2.5 Hz, 1H), 7.14 (dd, *J* = 8.9, 2.6 Hz, 1H), 6.76 (s, 2H), 3.86 (s, 3H); ^13^C NMR (150 MHz, DMSO-*d*_6_): *δ* 157.71, 139.11, 134.33, 132.45, 129.74, 128.92, 127.42, 125.29, 123.44, 119.14, 106.69, 55.65; HRMS (EI^+^, *m*/*z*): calcd for C_12_H_12_N_2_O^+^ [M]^+^: 200.0950, found 200.0947.

### 2.2 Preparation of stock solutions for Tl^3+^ detection

A 500 μM stock solution of probes TP-1 and TP-2 was prepared by dissolving in *N*,*N*-dimethylformamide (DMF). Stock solutions of metal ions and anions, each at a concentration of 10.0 mM, were prepared using deionized water (DI water). Tl^3+^ stock solution was prepared in 0.05 M HCl solution and standardized by iodometric titration.^41^ The acetate buffer solution, with a pH of 4.8, was formulated according to a procedure described in the literature.^42^

### 2.3 Preparation of samples for Tl^3+^ sensing

The sample solutions for Tl^3+^ signaling using TP-1 and TP-2 were prepared under optimized conditions using a pH 4.8 acetate buffer solution containing 1% (v/v) DMF. In a 15 mL sample vial, 15 μL of the 10.0 mM analyte stock solution was added and diluted with 2.80 mL of DI water and 150 μL of 200 mM acetate buffer solution at pH 4.8. Subsequently, 30 μL of a 0.50 mM solution of either probe TP-1 or TP-2 was added to the vial and gently mixed. The final concentrations of the probe, analyte, and buffer in the sample solution were 5.0 μM, 50 μM, and 10 mM, respectively. All samples were prepared and measured in triplicate, and the error bars were calculated based on the standard deviation of these measurements.

### 2.4 Exploring the mechanism of Tl^3+^ sensing of TP-1

To obtain the purified signaling product for Tl^3+^ sensing, probe TP-1 (0.021 g, 0.10 mmol) was dissolved in 10 mL of DMF. Thallium nitrate trihydrate (0.088 g, 0.20 mmol) was then dissolved in 0.50 mL of a 50 mM HCl solution. This thallium solution was subsequently diluted with 9.0 mL of DI water and 0.50 mL of 100 mM acetate buffer solution at pH 4.8. The thallium solution was carefully added to the probe TP-1 solution and mixed for 1 h. The reaction progression of the Tl^3+^ signaling was monitored using thin-layer chromatography (TLC) measurement. The resulting product was extracted with dichloromethane (DCM) and purified using column chromatography (silica gel, eluent: DCM). The purified product of Tl^3+^ sensing was then characterized by ^1^H and ^13^C NMR spectroscopy and mass spectrometry.

### 2.5 Tl^3+^ analysis of artificial urine samples

Tl^3+^ analysis of artificial urine samples using a smartphone as an all-in-one platform for signal acquisition and data processing was performed following a modified procedure described in the literature.^43^

#### 2.5.1 Preparation of sample solution

The possibility of Tl^3+^ level determination in urine samples was tested using commercially available artificial urine solution (Sigmatrix Urine Diluent). This solution is comprised of calcium chloride, magnesium chloride, potassium chloride, sodium phosphate monobasic, sodium sulfite, urea, creatinine, and sodium azide as an antimicrobial agent. In a 15 mL vial, the sample solution was prepared by adding 0.15 mL of 200 mM acetate buffer, 0.30 mL of artificial urine stock solution, and a calculated amount of Tl^3+^ ions (0–12 μL, 1.0 mM). The solution was then diluted with DI water to a final volume of 2.97 mL. Probe TP-1 (30 μL, 0.50 mM) was subsequently added to the mixture, followed by careful mixing. The final concentrations of TP-1, Tl^3+^ ion, and acetate buffer in the solution were 5.0 μM, 0–4.0 μM, and 10 mM, respectively.

#### 2.5.2 Tl^3+^ assay in artificial urine samples using a smartphone

To minimize interference from ambient light, a cube-shaped box with a square opening in the lid was designed to allow sample illumination using a handheld UV lamp (VILBER, VL-4LC). The prepared sample solution was added in a 3 mL of cuvette and placed at the center of the box. The images of the solution under UV irradiation were obtained using a smartphone (iPhone 15 pro, Apple) without any special settings. The fluorescence images were analyzed for their red, green, and blue channel intensities using a smartphone application (RGB Grabber, Shunamicode). Among the three channels, the blue channel exhibited the greatest variation, and a calibration curve was constructed by plotting its changes against [Tl^3+^] using a spreadsheet program embedded in the smartphone (Excel, Microsoft Corporation).

## 3. Results and discussion

**Scheme 1:**
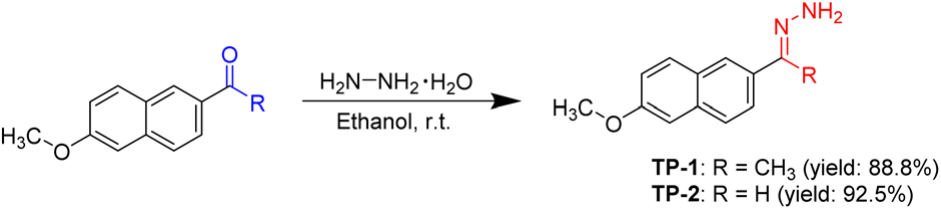
Preparation of Tl^3+^ signaling probes TP-1 and TP-2.

The Tl^3+^-selective probes have been developed using the unique oxidative properties of thallic ions, among other chemical reactions. For instance, oxidation of trifluoperazine derivative to its sulfoxide form,^21^ as well as the oxidative hydrolysis of sulfonhydrazine^24^ and rhodamine hydroxamate^25^ have been employed for successful Tl^3+^ analysis. In this study, we explored the Tl^3+^ signaling behavior of hydrazone derivatives of the methoxynaphthalene fluorophore, leveraging the unique Tl^3+^ metal ion-selective hydrolysis characteristic of the hydrazone moiety. The hydrolysis of hydrazones has previously been utilized for the construction of selective signaling probes targeting Cu^2+^ and Hg^2+^ ions.^34,37,44^ The designed probes, TP-1 and TP-2, were synthesized through the reaction of 2-acetyl-6-methoxynaphthalene (for ketone hydrazone) and 6-methoxy-2-naphthaldehyde (for aldehyde hydrazone) with hydrazine monohydrate in ethanol, yielding 88.8% and 92.5%, respectively (Scheme 1). The structures of probes TP-1 and TP-2 were confirmed by ^1^H and ^13^C NMR spectroscopy and high-resolution mass spectrometry (ESI†). Additionally, the photophysical properties of TP-1 and TP-2 in the presence and absence of Tl^3+^ ions are summarized in Table S2 (ESI).†

To investigate the Tl^3+^ signaling behaviors of TP-1 and TP-2, we initially examined the UV-vis spectra of both probes in the presence and absence of Tl^3+^ ions. As illustrated in Fig. S1 (ESI),† the absorbance changes of TP-1 and TP-2 were not significant. Consequently, we conducted the fluorescence-based Tl^3+^ signaling experiments. Ketone and aldehyde hydrazones have been reported to exhibit different stabilities and reactivities toward the target material.^37^ Therefore, we explored the time-dependent Tl^3+^ signaling behaviors of TP-1 and TP-2 and their stability under measurement conditions. As demonstrated in Fig. S2 (ESI),† the ketone-based hydrazone TP-1 exhibited rapid signaling in response to Tl^3+^ ions, and it remained stable without decomposing under the measurement conditions. In contrast, the aldehyde-based hydrazone TP-2 itself underwent hydrolysis under the experimental conditions (Fig. S3, ESI†). Consequently, the thallium signaling experiments were primarily conducted using the ketone-based hydrazone TP-1.

**Fig. 1:**
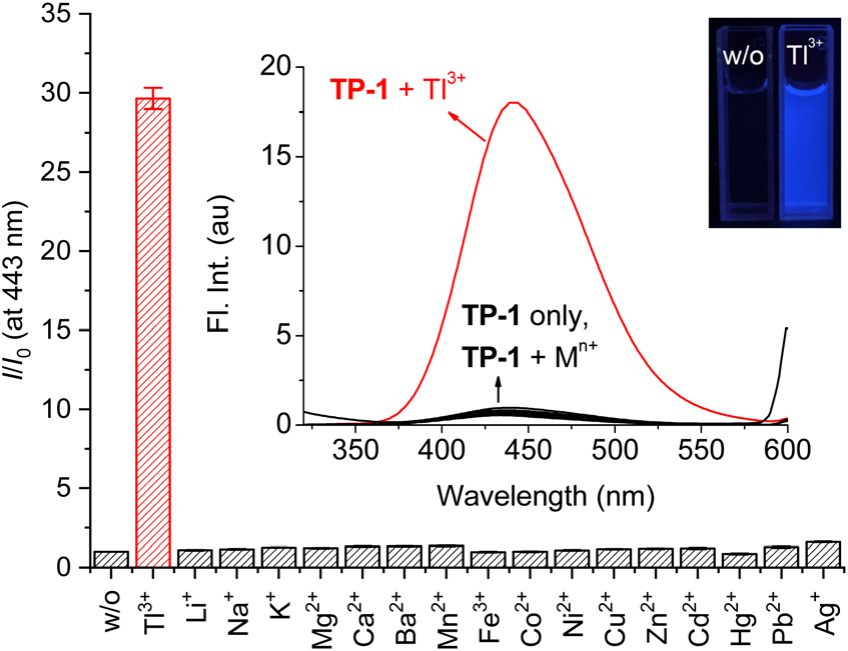
Changes in fluorescence intensity enhancement (*I*/*I*_0_) at 443 nm of TP-1 in the presence of common metal ions. Inset: fluorescence spectra and fluorescence image of TP-1. [TP-1] = 5.0 μM, [Tl^3+^] = [M^*n*+^] = 50 μM, in a pH 4.8 acetate buffer solution (10 mM) containing 1% (v/v) DMF. *λ*_ex_ = 309 nm.

Initially, we confirmed changes in the fluorescence emission of probe TP-1 in the presence of Tl^3+^ ions and other representative metal ions (Fig. 1). Probe TP-1 exhibited weak fluorescence emission around 435 nm. However, upon treatment with Tl^3+^ ion, the probe displayed a significant enhancement in fluorescence emission at 443 nm, along with blue fluorescence under UV-lamp irradiation. Conversely, the other tested metal ions did not exhibit any noticeable changes. We quantified these fluorescence changes of TP-1 with metal ions by measuring the fluorescence enhancement at 443 nm (*I*/*I*_0_ at 443 nm). As depicted in Fig. 1, the fluorescence enhancement (*I*/*I*_0_) for Tl^3+^ ions was 29.6. Meanwhile, the *I*/*I*_0_ values for other metal ions varied within a narrow range, from 0.86 for Hg^2+^ ions to 1.61 for Ag^+^ ions. Additionally, we investigated the fluorescence signaling behavior of TP-1 toward common anions. As shown in Fig. S4 (ESI),† probe TP-1 demonstrated a significant fluorescence response toward Tl^3+^ ions over the tested anions, with the fluorescence enhancement (*I*/*I*_0_) ranging narrowly from 0.93 for SO_3_^2−^ to 1.81 for P_2_O_7_^4−^.

**Fig. 2:**
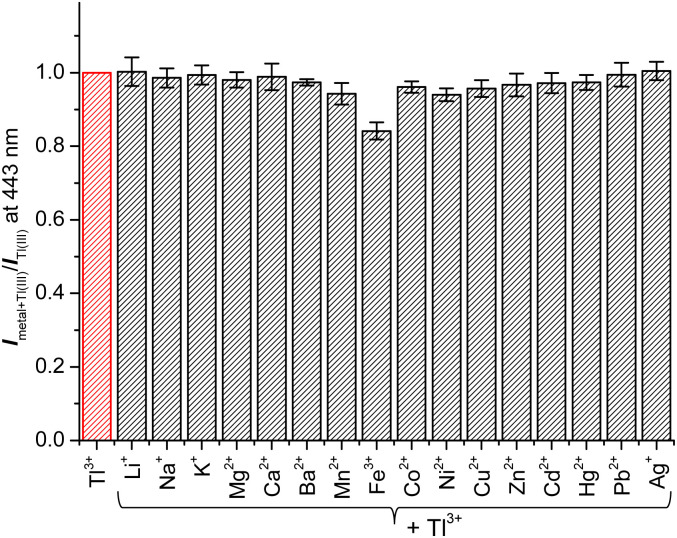
Changes in fluorescence intensity ratio (*I*_metal+Tl(iii)_/*I*_Tl(iii)_) of TP-1 at 443 nm in the presence of coexisting metal ions. [TP-1] = 5.0 μM, [Tl^3+^] = [M^*n*+^] = 50 μM, in a pH 4.8 acetate buffer solution (10 mM) containing 1% (v/v) DMF. *λ*_ex_ = 309 nm.

Next, to assess the practical applicability of TP-1 for sensing Tl^3+^ ions in urine, we evaluated the effect of coexisting ions on the Tl^3+^ signaling behavior of the probe. As illustrated in Fig. 2, the signaling of the probe for Tl^3+^ was unaffected by the presence of other tested metal ions serving as background. The fluorescence intensity enhancement ratio (*I*_metal+Tl(iii)_/*I*_Tl(iii)_) of TP-1 in the presence of common metal ions varied slightly, ranging from 84.2% for Fe^3+^ to 100.5% for Ag^+^. Additionally, we verified that the Tl^3+^ signaling of the probe was not impacted by common background anionic species (Fig. S5, ESI†). The fluorescence intensity enhancement ratio (*I*_anion+Tl(iii)_/*I*_Tl(iii)_) for the tested anions fluctuated minimally, from 93.2% for I^−^ to 100.1% for F^−^. Furthermore, we tested the fluorescence response of the probe toward representative urine components such as uric acid, urea, and creatinine for its application to the urinary thallium determination.^45^ As shown in Fig. S6 (ESI),† in the presence of these urine components, the Tl^3+^ signaling behavior was not affected noticeably. Furthermore, we confirmed that the Tl^3+^ signaling of TP-1 remained stable in their presence.

**Scheme 2:**
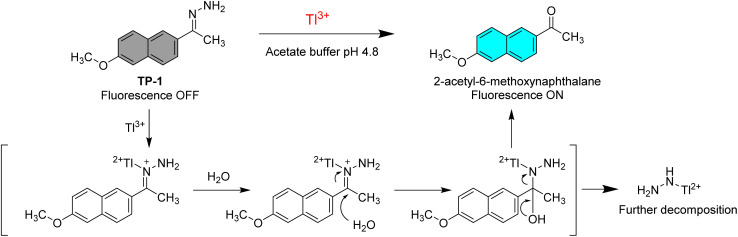
Suggested Tl^3+^ sensing mechanism of TP-1.

**Fig. 3:**
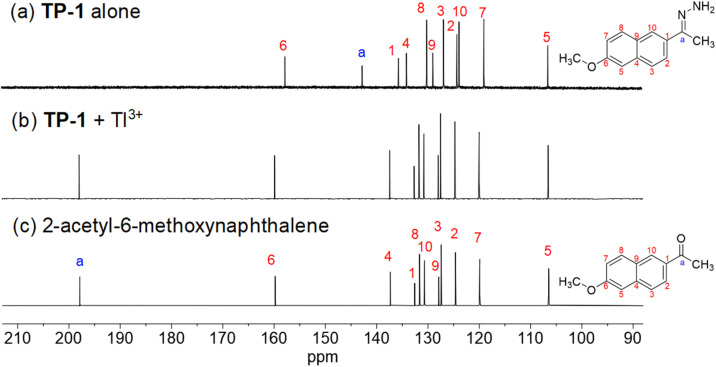
Partial ^13^C NMR spectra of (a) TP-1, (b) TP-1 + Tl^3+^, and (c) 2-acetyl-6-methoxynaphthalene in DMSO-*d*_6_. [TP-1] = [2-acetyl-6-methoxynaphthalene] = 5.0 mM. For (b), the spectrum (TP-1 + Tl^3+^) was obtained using a purified product of a mixture of TP-1 (5.0 mM) and Tl(NO_3_)_3_ (10.0 mM) in acetate buffer (pH 4.8) containing 1% (v/v) DMF.

The Tl^3+^ signaling mechanism of TP-1 can be explained by the Tl^3+^-assisted hydrolysis of the hydrazone moiety of the probe, yielding the strongly fluorescent 2-acetyl-6-methoxynaphthalene fluorophore (Scheme 2). This proposed sensing mechanism was investigated through ^1^H/^13^C NMR and mass measurements, as well as TLC monitoring of the Tl^3+^ sensing process. In the ^13^C NMR spectra, probe TP-1 displayed a hydrazone C=N carbon peak at 142.6 ppm. However, after treatment with Tl^3+^ ions, the C=N carbon peak disappeared, and a new carbonyl C=O carbon peak at 197.9 ppm appeared (Fig. 3). Additionally, as shown in the ^1^H NMR spectra (Fig. S7, ESI†), we confirmed that the hydrazone NH_2_ protons of the probe at 6.38 ppm completely disappeared following treatment with Tl^3+^ ions. We also confirmed that the ^1^H and ^13^C NMR spectra of the signaling product of the probe were identical to those of the expected signaling product, 2-acetyl-6-methoxynaphthalene. Furthermore, from the mass analysis, we confirmed that the Tl^3+^ signaling product of the probe exhibited a peak with *m*/*z* = 200.1, consistent with 2-acetyl-6-methoxynaphthalene (calculated *m*/*z* = 200.08) (Fig. S8, ESI†). In addition, TLC monitoring also ascertained that probe TP-1 yielded the strongly fluorescent 2-acetyl-6-methoxynaphthalene as a signaling product (Fig. S9, ESI†). Moreover, to further investigate the sensing mechanism, we conducted pH-dependent Tl^3+^ signaling studies. As shown in Fig. S10 (ESI),† the fluorescence signaling of TP-1 for Tl^3+^ ions decreased dramatically below pH 3.0. This result is attributed to the protonation of the hydrazone moiety, which disrupts Tl^3+^ binding and inhibits the hydrolysis mechanism of the signaling process.

**Fig. 4:**
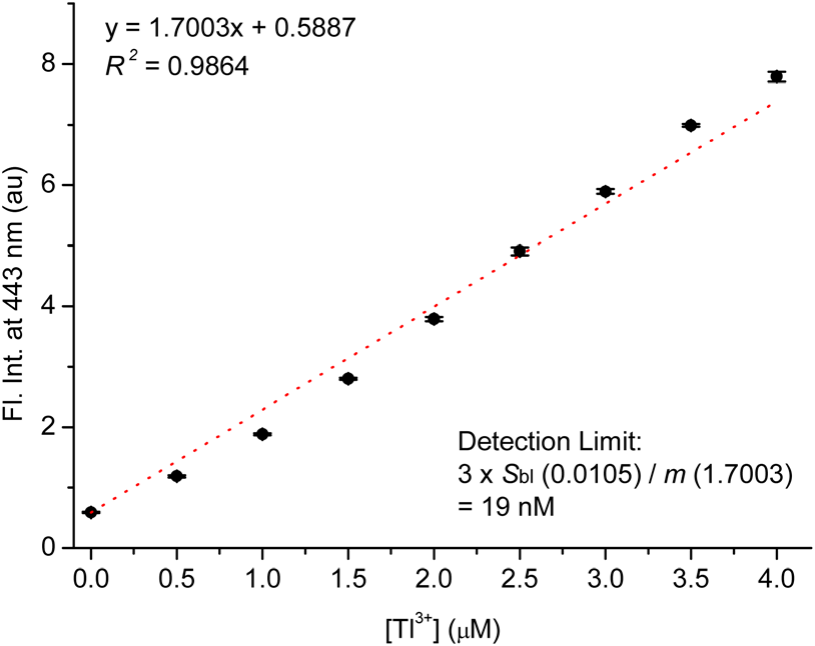
Calibration curve for fluorescence titration of TP-1 with Tl^3+^. [TP-1] = 5.0 μM, [Tl^3+^] = 0–4.0 μM, in a pH 4.8 acetate buffer solution (10 mM) containing 1% (v/v) DMF. *λ*_ex_ = 309 nm.

To estimate the detection limit of TP-1 for Tl^3+^ ions, we conducted experiments to observe the Tl^3+^ concentration-dependent fluorescence signaling behavior of the probe (Fig. 4). The fluorescence emission at 443 nm linearly increased with increasing concentrations of Tl^3+^ (*R*^2^ = 0.9864). The detection limit for Tl^3+^ was determined following the IUPAC recommendation, using the equation LOD = 3*s*_blk_/*m*, where *s*_blk_ represents the standard deviation of the blank signal and *m* denotes the slope of the calibration curve.^46^ From Fig. 4, the standard deviation of the blank signal (*s*_blk_) was calculated to be 0.0105, and the slope of the titration curve (*m*) was determined to be 1.7003. Using these values, the detection limit for Tl^3+^ was calculated to be 19 nM. This low detection limit highlights the high sensitivity of TP-1 for Tl^3+^ detection, making it suitable for practical applications such as clinical diagnostics and environmental monitoring. Next, we confirmed the pH profile of the Tl^3+^ signaling across a pH range from 1.3 to 7.0. As shown in Fig. S10 (ESI),† Tl^3+^ signaling of the probe was effective in the pH range from 4.8 to 7.0. In acidic conditions, TP-1 exhibited an increase in fluorescence due to the protonation of the hydrazone moiety. However, the Tl^3+^ signaling of TP-1 decreased markedly under highly acidic conditions because the protonation disrupted Tl^3+^ binding at the hydrazone moiety, thereby interfering the signaling process. Meanwhile, in basic conditions, Tl^3+^ signaling slightly decreased because Tl^3+^ ions can be converted to Tl_2_O_3_.^47^ Additionally, we evaluated the stability of TP-1 stock solutions under different storage conditions. The Tl^3+^ response of TP-1 was tested using stock solutions stored for immediate use, 1 day, 3 days, 5 days, and 1 week at room temperature in the dark. As shown in Fig. S11 (ESI),† the results confirmed that TP-1 retains its fluorescence signaling ability throughout these storage periods, demonstrating its stability and suitability for practical applications.

**Fig. 5:**
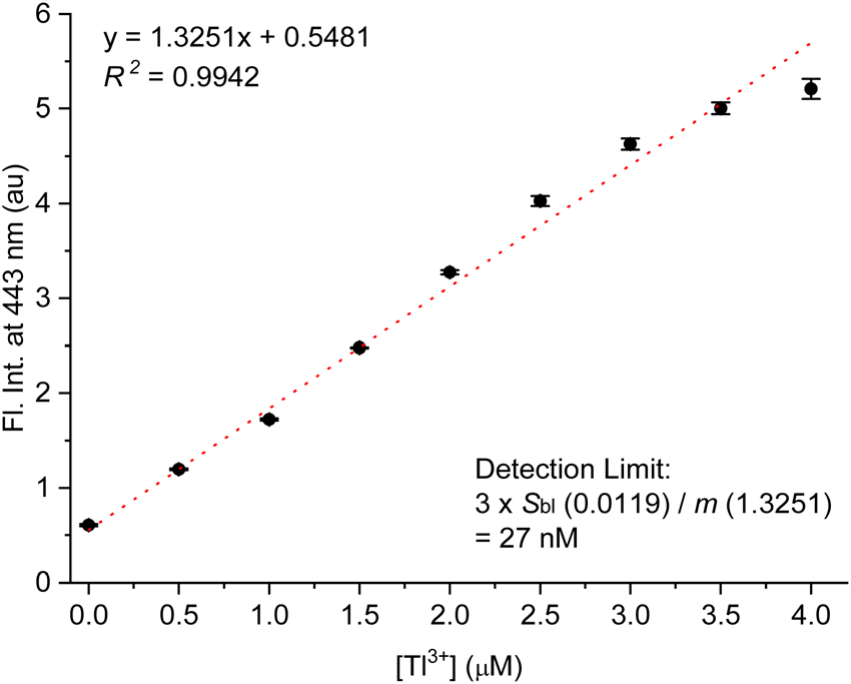
Concentration-dependent Tl^3+^ signaling of TP-1 in artificial urine. [TP-1] = 5.0 μM, [Tl^3+^] = 0–4 μM, [buffer] = 10 mM in a pH 4.8 acetate buffered artificial urine solution containing 1% (v/v) DMF. *λ*_ex_ = 309 nm.

As mentioned in the introduction, urinary thallium is one of the most convenient indicators for diagnosing thallium poisoning, alongside testing thallium ions in hair and blood samples. For this reason, we investigated the Tl^3+^ signaling of TP-1 in artificial urine samples. As shown in the previous results, we evaluated the interference effects of representative urine components, including urea, creatinine, uric acid, common metal ions and anions, ammonia, and glucose. These tests confirmed that the assay components do not produce background fluorescence or interfere with the Tl^3+^ signaling of TP-1. Therefore, we examined the Tl^3+^ concentration-based fluorescence signaling behavior in artificial urine solutions using the fluorescence spectroscopy. As shown in Fig. 5, the fluorescence intensity at 443 nm quantitatively increased with the Tl^3+^ concentration up to 4.0 μM (*R*^2^ = 0.9942). This result demonstrates that TP-1 can reliably determine Tl^3+^ ions in artificial urine samples.

**Fig. 6:**
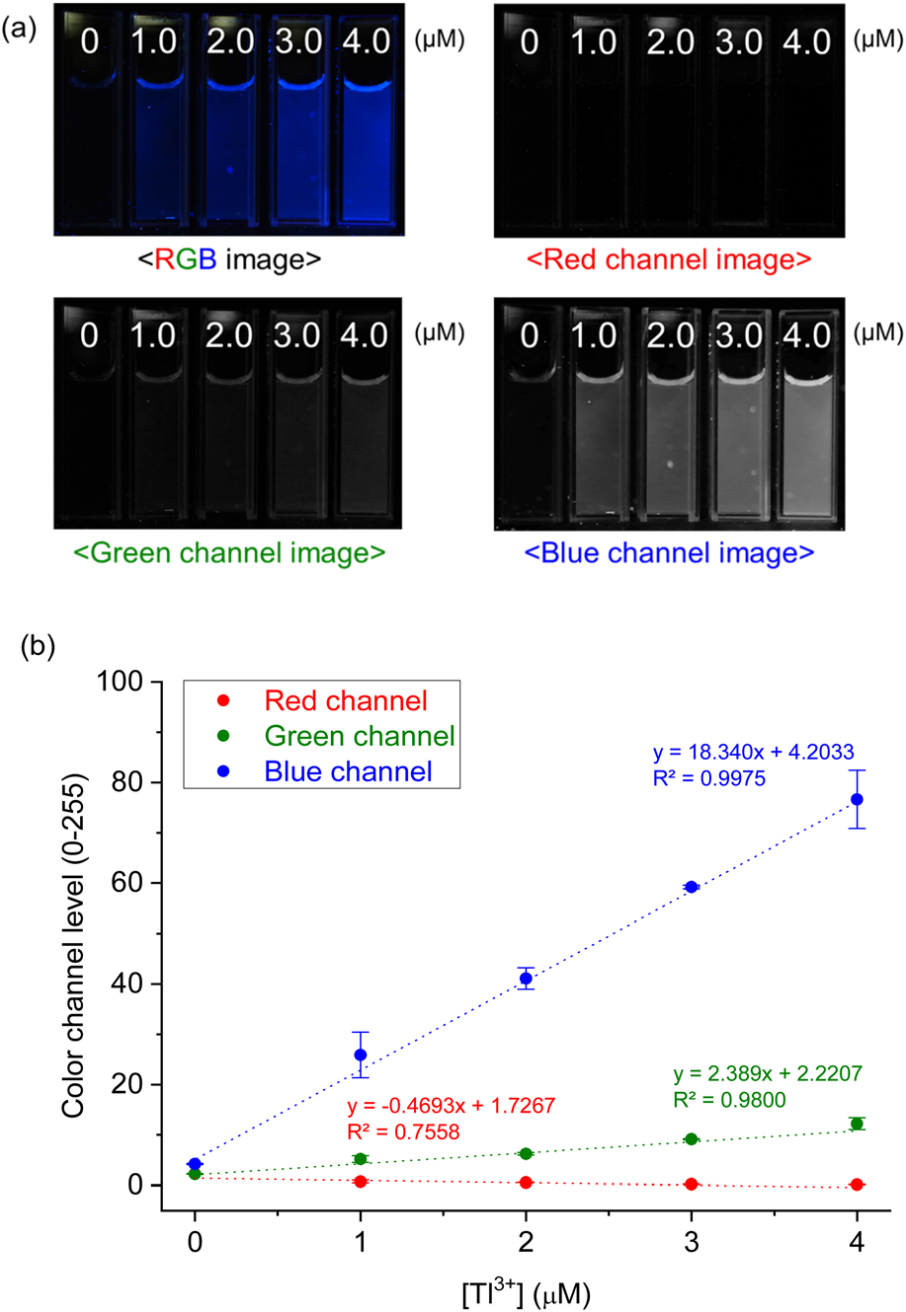
(a) Fluorescence and its color filtered (RGB) images of TP-1 as increased of [Tl^3+^] in urine solution. (b) Calibration curve of the color channel level versus [Tl^3+^] concentration. [TP-1] = 5.0 μM, [Tl^3+^] = 0–4.0 μM, [buffer] = 10 mM in artificial urine solution containing 1% (v/v) DMF.

Next, to evaluate the practical applicability of the probe, a Tl^3+^ assay in artificial urine samples was conducted using a smartphone as an easily accessible device for image capture and data analysis.^48^ As demonstrated in Fig. 6a, TP-1 exhibited enhanced blue fluorescence with increasing Tl^3+^ concentrations. The RGB color channel levels of the fluorescence images were analyzed using a smartphone-based color analysis application. A calibration curve based on the blue channel showed satisfactory linearity for Tl^3+^ ions in artificial urine samples (Fig. 6b). Although the assay showed a relatively high error of 18.0% at a low concentration of 1.0 μM, the smartphone-based results were in good agreement with those obtained from fluorescence method (Table 1). While traditional methods using a fluorescence spectroscopy are generally recognized for their superior accuracy and precision, the smartphone-based method provides a practical and rapid alternative for detecting Tl^3+^ ions. This approach is particularly advantageous for field settings and point-of-care applications, where accessibility and simplicity are critical. The smartphone-based assay demonstrated reliable performance useful for rapid screening in suspected cases of acute thallium poisoning, making it a valuable tool for on-site diagnostics. From these findings, we conclude that TP-1 is a robust probe for detecting Tl^3+^ ions in artificial urine, offering a rapid, cost-effective, and accessible method for on-site Tl^3+^ detection. While this study focused on artificial urine to mimic real-world conditions due to ethical constraints, the results strongly support the practical applicability of TP-1 for diagnosing acute Tl^3+^ poisoning in complex biological matrices.

**Table 1:** Assay of Tl^3+^ ions in artificial urine solution using a smartphone and fluorescence spectroscopy^a^

[Tl^3+^] (μM)^b^	Using smartphone (μM)	Relative error (%)	Using fluorescence spectroscopy (μM)	Relative error (%)
0	Not detected	—	Not detected	—
1.0	1.18 ± 0.26	18.0	0.89 ± 0.09	−11.3
2.0	2.01 ± 0.12	−0.5	2.05 ± 0.02	2.8
3.0	2.99 ± 0.02	−0.5	3.07 ± 0.05	2.6

a Reported values are given as mean ± standard deviation, *n* = 3.

b [Tl^3+^] was standardized using iodometric titration.

## 4. Conclusions

We have developed a convenient Tl^3+^-selective fluorescent signaling probe, which is simple to prepare and effective for the early diagnosis of acute thallium poisoning using easily available urine samples. Probe TP-1, a hydrazone derivative of 2-acetyl-6-methoxynaphthalene, demonstrated pronounced turn-on type fluorescence signaling behavior specifically in response to Tl^3+^ ions. The mechanism of Tl^3+^ signaling of the probe involves the unique Tl^3+^-assisted hydrolysis of the hydrazone moiety, leading to the generation of the parent fluorophore. Notably, the Tl^3+^ signaling was unaffected by the presence of common metal ions, anions, and major urine components such as uric acid, urea, and creatinine. Furthermore, structural improvement through comparative testing of ketone-hydrazone (TP-1) and aldehyde-hydrazone (TP-2) derivatives confirmed that probe TP-1 exhibited rapid and stable Tl^3+^ signaling behavior with sufficient stability under the measurement conditions. The utility of the probe was further highlighted by successfully conducting a Tl^3+^ assay in artificial urine samples using a smartphone for signal capture and analysis. These results affirm the practical potential of probe TP-1 for diagnosing acute thallium poisoning in readily obtainable human urine samples.

## Data availability

Data will be made available upon request.

## Conflicts of interest

There are no conflicts of interest to declare.

## Supplementary Material

RA-015-D4RA06726F-s001
