# Supplementary material for: Selective fluorescent probe for Tl3+ ions through metal-induced hydrolysis and its application for direct assay of artificial urine
Source: RSC Adv. 2025 Jan 15;15(2):1336–43. doi: 10.1039/d4ra06726f (PMC11734399; doi:10.1039/d4ra06726f)
Supplement: RA-015-D4RA06726F-s001 [file RA-015-D4RA06726F-s001.pdf]

Supplementary Information (SI) for RSC Advances.
This journal is © The Royal Society of Chemistry 2025

Electronic Supplementary Information

Selective fluorescent probe for Tl^{3+} ions through metal-induced hydrolysis and its application for direct assay of artificial urine

Myung Gil Choi, Yerin Jang, Mi Gang Kim, Sangdoo Ahn* and Suk-Kyu Chang*

Department of Chemistry, Chung-Ang University, Seoul 06974, Republic of Korea

Contents

Experimental details

Table S1. Representative Tl^{3+} signaling sensors and reaction-based probes reported previously

Table S2. Photophysical properties of **TP-1** and **TP-2** in the presence and absence of Tl^{3+} ions

Fig. S1. UV–vis spectra of (a) **TP-1** and (b) **TP-2** in the presence and absence of Tl^{3+}.

Fig. S2. Time-dependent fluorescence emission of **TP-1** at 443 nm in response to Tl^{3+}.

Fig. S3. Time-dependent fluorescence emission of **TP-2** at 455 nm in response to Tl^{3+}.

Fig. S4. Changes in fluorescence intensity enhancement (I/I_0) at 443 nm of **TP-1** in the presence of common anions. Inset: fluorescence spectra of **TP-1**.

Fig. S5. Changes in fluorescence intensity ratio ($I_{anion+Tl(III)}/I_{Tl(III)}$) of **TP-1** at 443 nm in the presence of coexisting anions.

Fig. S6. Changes in fluorescence intensity enhancement (I/I_0) of **TP-1** at 443 nm in the presence of urine components.

Fig. S7. Partial ^1H NMR spectra of (a) **TP-1**, (b) **TP-1** + Tl^{3+}, and (c) 2-acetyl-6-methoxynaphthalene in DMSO-d_6.

Fig. S8. Mass spectrum of the Tl^{3+} signaling product of **TP-1**.

Fig. S9. TLC profile of **TP-1** (spot A), **TP-1** with Tl^{3+} (2.0 eq.) (spot B), and 2-acetyl-6-methoxynaphthalene (spot C).

Fig. S10. Effect of pH on the Tl^{3+} signaling of **TP-1** expressed by changes in fluorescence intensity at 443 nm.

Fig. S11. Stability of **TP-1** stock solution over different storage durations.

Fig. S12. ^{1}H NMR spectrum of **TP-1** in DMSO-d_6 (600 MHz).

Fig. S13. ^{13}C NMR spectrum of **TP-1** in DMSO-d_6 (150 MHz).

Fig. S14. HSQC NMR spectrum of **TP-1** in DMSO-d_6.

Fig. S15. HMBC NMR spectrum of **TP-1** in DMSO-d_6.

Fig. S16. High-resolution mass spectrum of **TP-1**.

Fig. S17. HPLC chromatogram of **TP-1**.

Fig. S18. ^{1}H NMR spectrum of **TP-2** in DMSO-d_6 (600 MHz).

Fig. S19. ^{13}C NMR spectrum of **TP-2** in DMSO-d_6 (150 MHz).

Fig. S20. High-resolution mass spectrum of **TP-2**.

Fig. S21. HPLC chromatogram of **TP-2**.

Experimental details

1. General

Fluorophores 2-acetyl-6-methoxynaphthalene and 6-methoxy-2-naphthaldehyde were purchased from Tokyo Chemical Industry Co. (TCI). Hydrazine monohydrate was obtained from Yakuri Pure Chemicals Co. NMR (^{1}H and ^{13}C) spectra were measured using a Varian VNS NMR spectrometer. High-resolution mass spectrometry (HRMS) was conducted using a JEOL JMS-700 mass spectrometer with electron ionization (EI). UV–vis and fluorescence spectra were measured using Scinco S-3100 and FS-2 spectrophotometers, respectively.

Table S1. Representative Tl^{3+} signaling sensors and reaction-based probes reported previously

Structure	Signal	Conditions	Mechanism	LOD	Application	Merit	Ref.
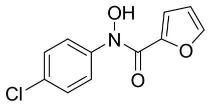	Colorimetry	pH 7.0 aqueous solution with 0.1% acetonitrile	Tl^{3+}–hydroxamic acid complex formation	-	Tl^{3+} assay in standard samples	● Naked eye detection	S1
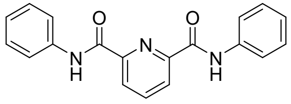	Colorimetry	Glycine-HCl buffer (pH 1.0)	Tl^{3+}-2,6-bis(*N*-phenyl carbamoyl) pyridine complex formation	1.2 nM (pre-concentration)	Tl^{3+} assay in environmental and biological samples	● Naked eye detection<br>● High sensitivity	S2
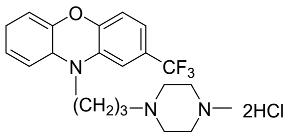	Colorimetry	H_2PO_4 solution	Oxidation of trifluoperazine	26,000 nM	Tl^{3+} assay in environmental and biological samples	● Naked eye detection<br>● Biological application	S3
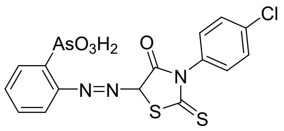	Fluorescence	Acetone containing 10% HCl	Oxidation of arsenoxylphenylazo rhodanine	0.0013 nM (pre-concentration)	Tl^{3+} assay in environmental samples	● High sensitivity	S4

Table S1. Representative Tl^{3+} signaling sensors and reaction-based probes reported previously (*continued*)

Structure	Signal	Conditions	Mechanism	LOD	Application	Merit	Ref.
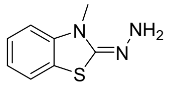	Colorimetry	H_2PO_4 solution	Oxidative coupling of **MBTH** and **IDH**	72,000 nM	Tl^{3+} assay in environmental and biological samples	● Naked eye detection ● Biological application	S5
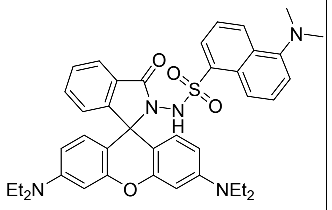	Colorimetry, Fluorescence	pH 4.76 acetate buffer with 20% (*v/v*) DMSO	Hydrolysis of sulfonhydrazide	190 nM	Tl^{3+} assay in commercial reagent using office scanner	● Naked eye detection ● IT-device-based technique	S6
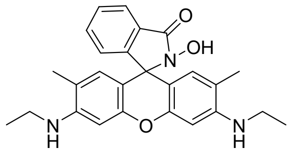	Colorimetry, Fluorescence	pH 4.2 acetate buffer with 30% (*v/v*) DMSO	Hydrolysis of hydroxamate	290 nM	Tl^{3+} assay in biological samples using smartphone	● Naked eye detection ● Biological application ● IT-device-based technique	S7
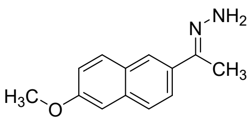	Fluorescence	pH 4.8 acetate buffer with 1% (*v/v*) DMF	Hydrolysis of hydrazone	19 nM	Tl^{3+} assay in biological samples using smartphone	● High sensitivity ● Biological application ● IT-device-based technique	This work

Table S2. Photophysical properties of **TP-1** and **TP-2** in the presence and absence of Tl^{3+} ions

	**Maximum λ_{abs} (nm)**	**Molar extinction coefficient (ε) ($cm^{-1}M^{-1}$)**	**Maximum λ_{em} (nm)**	**Quantum yield (Φ)[a]**
**TP-1** only	292	14,704	435	0.011
**TP-1** + Ti^{3+}	310	11,256	443	0.33
**TP-2** only	306	19,682	436	0.015
**TP-2** + Ti^{3+}	315	10,974	455	0.20

[a] Fluorescence quantum yields were measured by comparing with anthracene as a reference ($\Phi_{anthracene}$ = 0.27, ethanol).[S8]

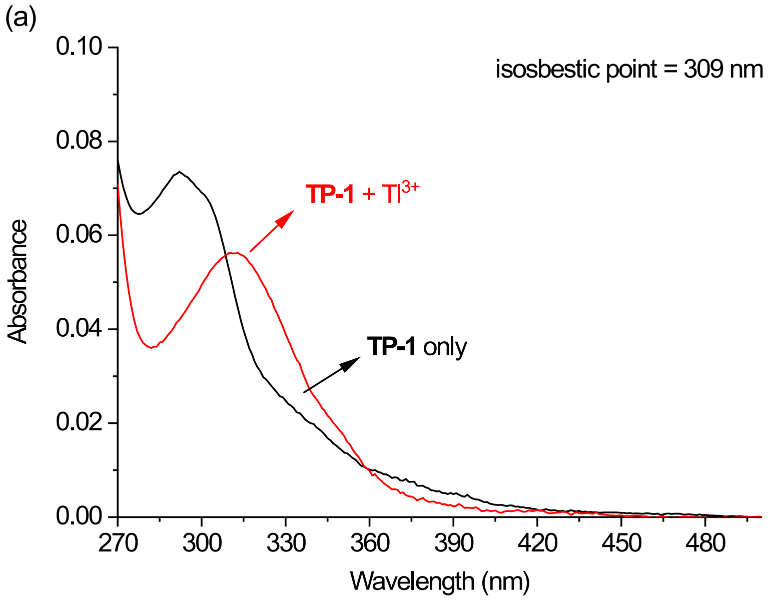

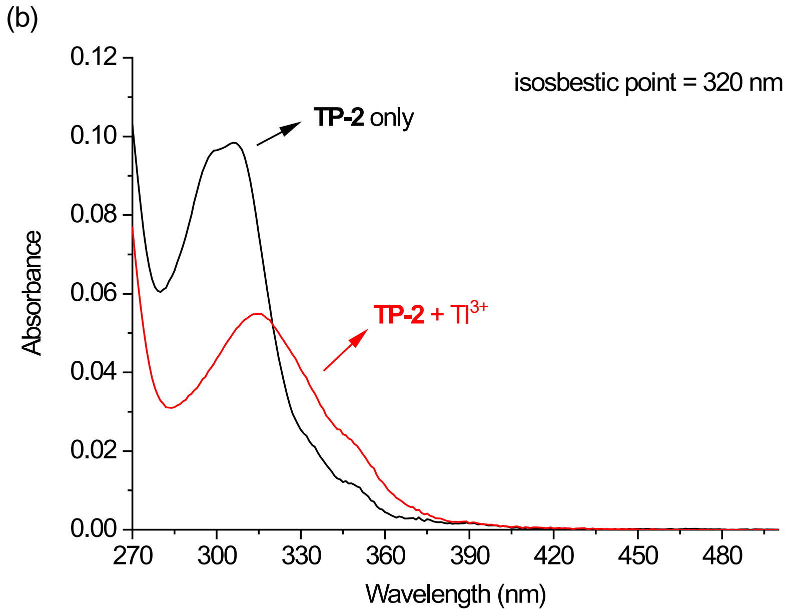

Fig. S1. UV–vis spectra of (**a**) **TP-1** and (**b**) **TP-2** in the presence and absence of Tl^{3+}. [**TP-1**] = [**TP-2**] = 5.0 μM, [Tl^{3+}] = 50 μM, in a pH 4.8 acetate buffer solution (10 mM) containing 1% (*v/v*) DMF.

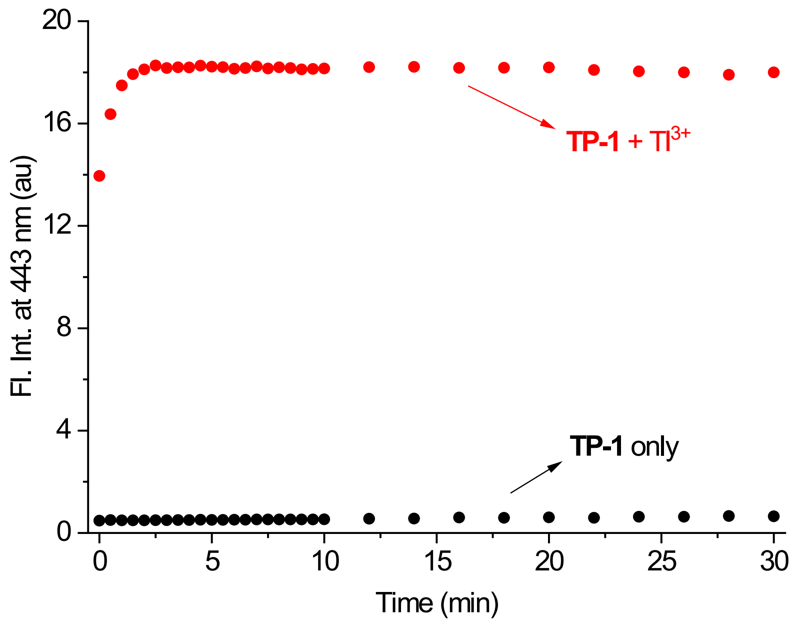

Fig. S2. Time-dependent fluorescence emission of **TP-1** at 443 nm in response to Tl^{3+}. [**TP-1**] = 5.0 μM, [Tl^{3+}] = 50 μM, in a pH 4.8 acetate buffer solution (10 mM) containing 1% (*v*/*v*) DMF. λ_{ex} = 309 nm.

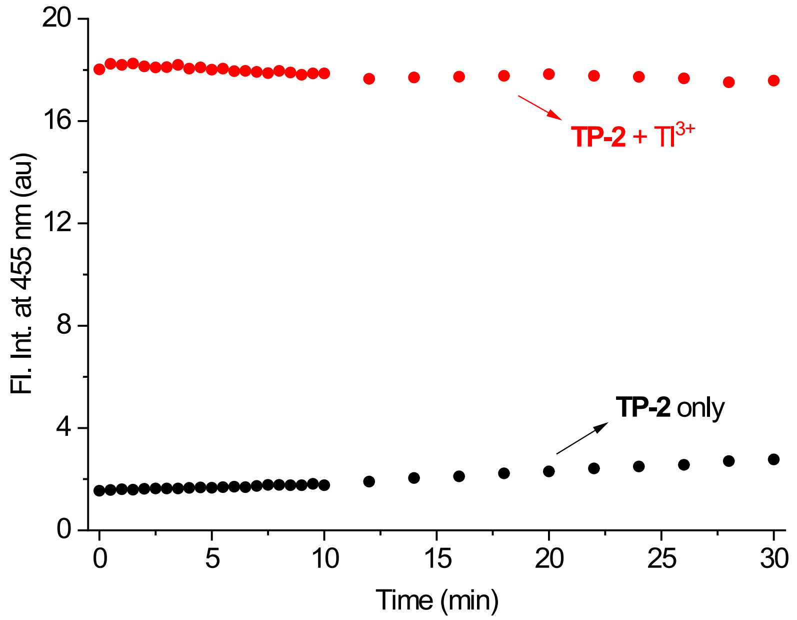

Fig. S3. Time-dependent fluorescence emission of **TP-2** at 455 nm in response to Tl^{3+}. [**TP-2**] = 5.0 μM, [Tl^{3+}] = 50 μM, in a pH 4.8 acetate buffer solution (10 mM) containing 1% (*v*/*v*) DMF. λ_{ex} = 320 nm.

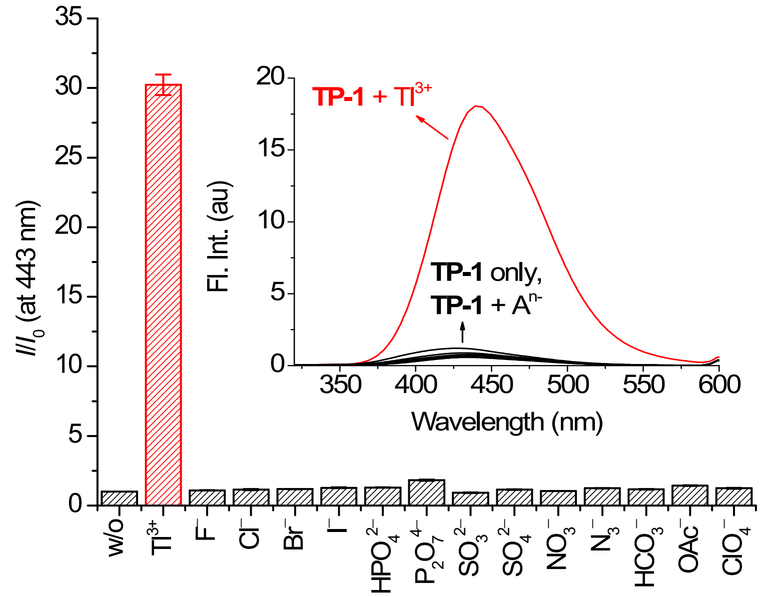

Fig. S4. Changes in fluorescence intensity enhancement (I/I_0) at 443 nm of **TP-1** in the presence of common anions. Inset: fluorescence spectra of **TP-1**. [**TP-1**] = 5.0 μM, [Tl^{3+}] = [A^{n-}] = 50 μM, in a pH 4.8 acetate buffer solution (10 mM) containing 1% (*v/v*) DMF. λ_{ex} = 309 nm.

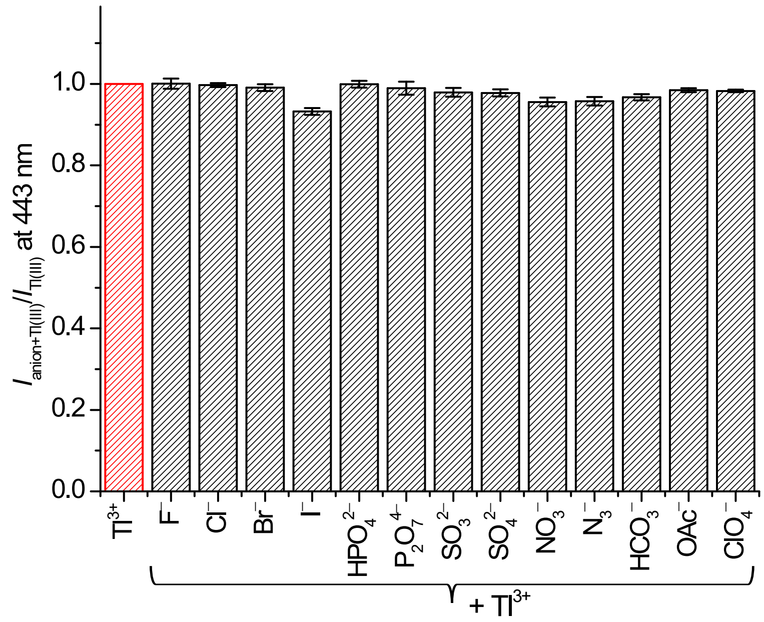

Fig. S5. Changes in fluorescence intensity ratio ($I_{anion+Tl(III)}/I_{Tl(III)}$) of **TP-1** at 443 nm in the presence of coexisting anions. [**TP-1**] = 5.0 μM, [Tl^{3+}] = [A^{n-}] = 50 μM, in a pH 4.8 acetate buffer solution (10 mM) containing 1% (*v/v*) DMF. λ_{ex} = 309 nm.

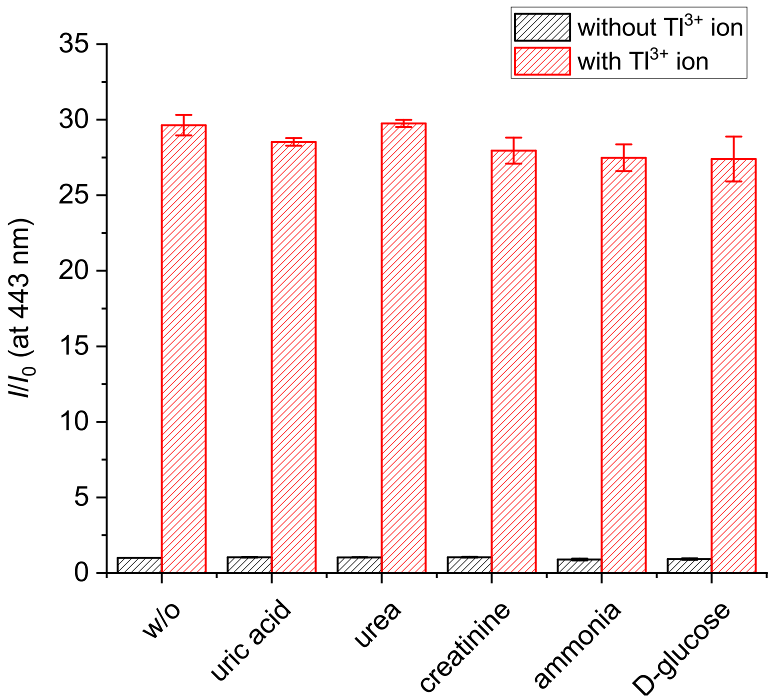

Fig. S6. Changes in fluorescence intensity enhancement (I/I_0) of **TP-1** at 443 nm in the presence of urine components. [**TP-1**] = 5.0 µM, [Tl^{3+}] = [urine component] = 50 µM, in a pH 4.8 acetate buffer solution (10 mM) containing 1% (*v/v*) DMF. λ_{ex} = 309 nm.

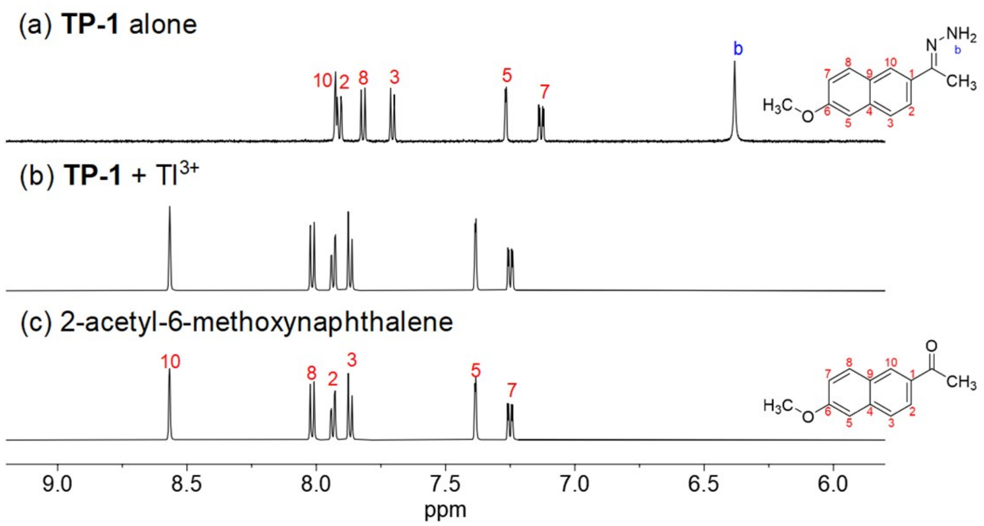

Fig. S7. Partial ^{1}H NMR spectra of (**a**) **TP-1**, (**b**) **TP-1** + Tl^{3+}, and (**c**) 2-acetyl-6-methoxynaphthalene in DMSO-d_6. [**TP-1**] = [2-acetyl-6-methoxynaphthalene] = 5.0 mM. For (b), the spectrum (**TP-1** + Tl^{3+}) was obtained using a purified product of a mixture of **TP-1** (5.0 mM) and $Tl(NO_3)_3$ (10.0 mM) in acetate buffer solution (pH 4.8) containing 1% (*v/v*) DMF.

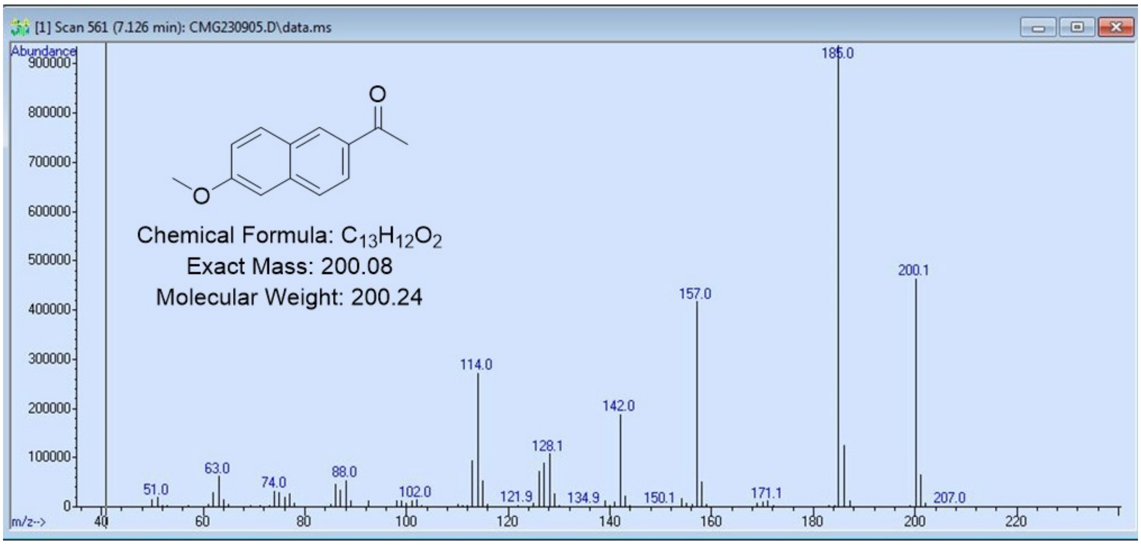

Fig. S8. Mass spectrum of the Tl^{3+} signaling product of **TP-1**.

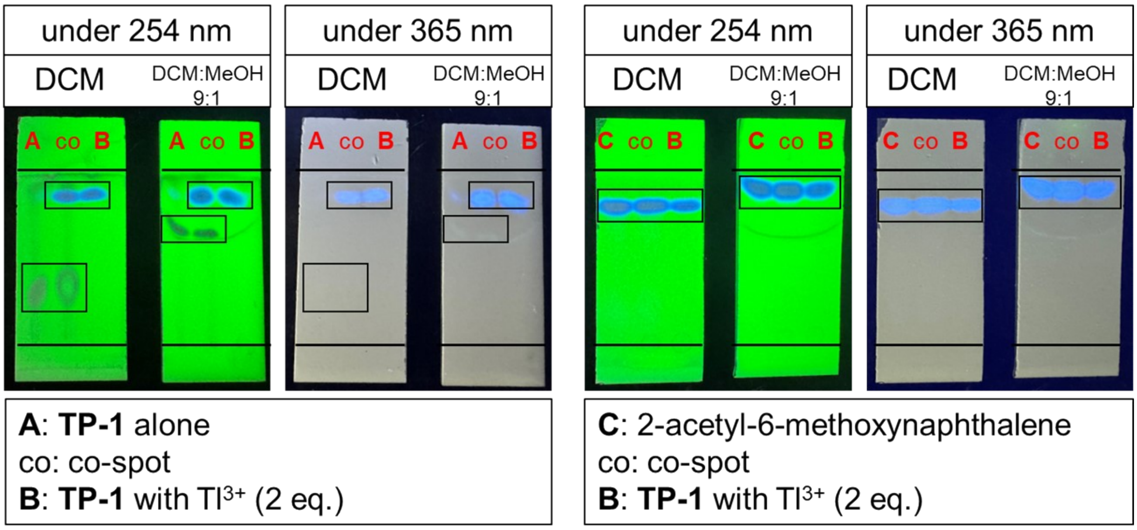

Fig. S9. TLC profile of **TP-1** (spot A), **TP-1** with Tl^{3+} (2.0 eq.) (spot B), and 2-acetyl-6-methoxynaphthalene (spot C).

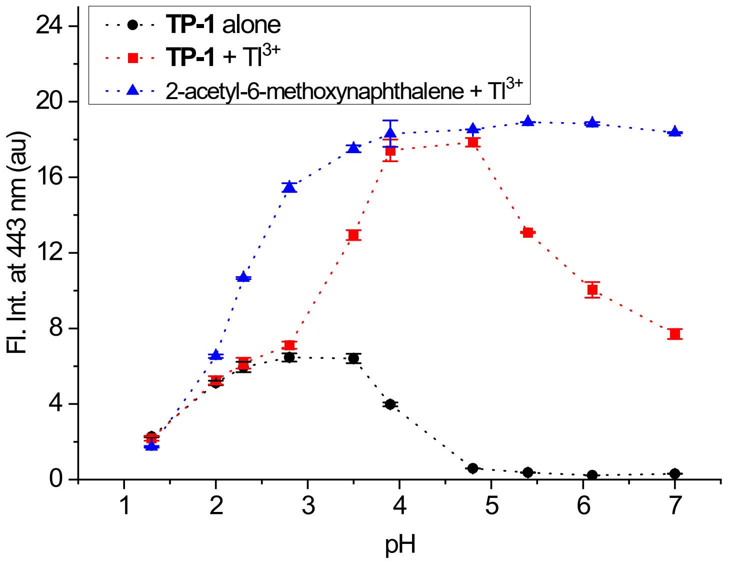

Fig. S10. Effect of pH on the Tl^{3+} signaling of **TP-1** expressed by changes in fluorescence intensity at 443 nm. [**TP-1**] = [2-acetyl-6-methoxynaphthalene] = 5.0 μM, [Tl^{3+}] = 50 μM, in a pH 4.8 acetate buffer solution (10 mM) containing 1% (*v/v*) DMF. HCl or NaOH solution was added to adjust the pH. λ_{ex} = 309 nm.

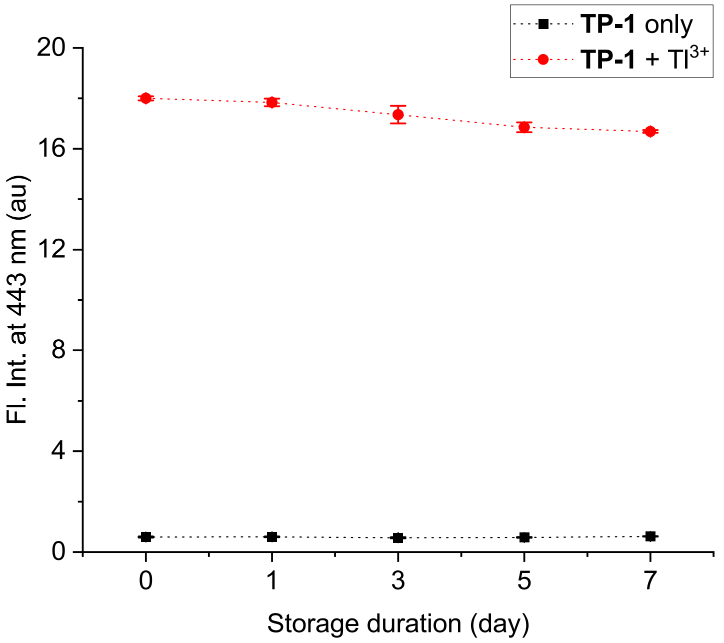

Fig. S11. Stability of **TP-1** stock solution over different storage durations. [**TP-1**] = 5.0 μM, [Tl^{3+}] = 50 μM, in a pH 4.8 acetate buffer solution (10 mM) containing 1% (*v/v*) DMF. The stock solutions were used after being stored for immediate use, 1 day, 3 days, 5 days, and 1 week at room temperature in the dark. λ_{ex} = 309 nm.

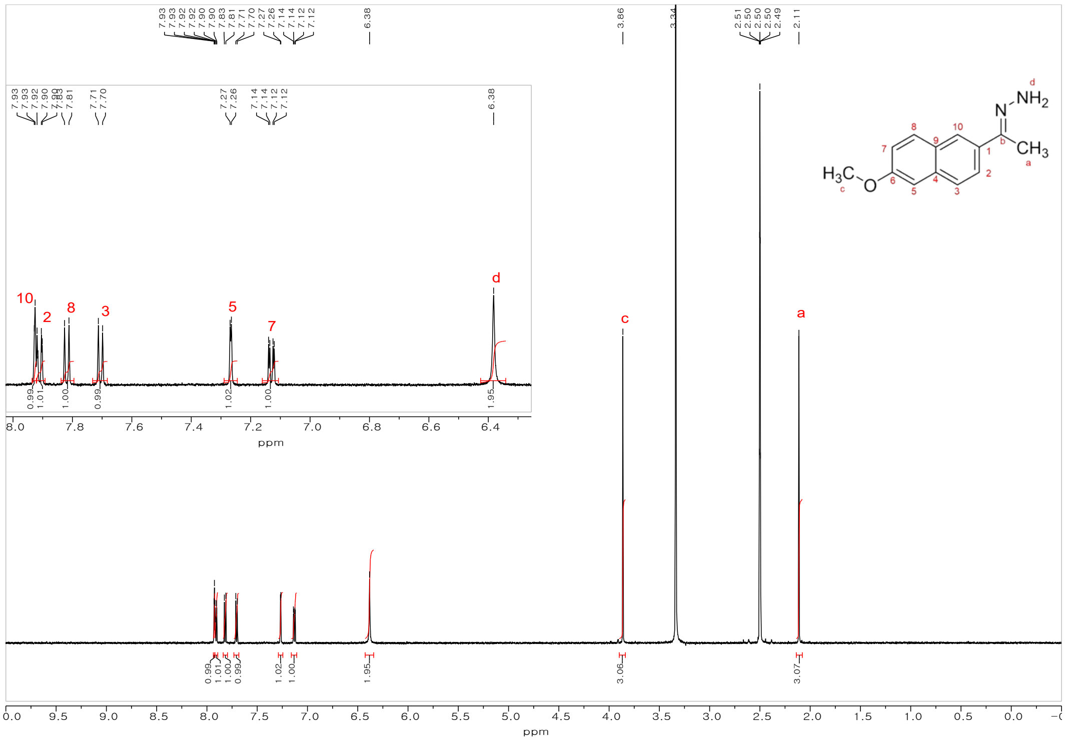

Fig. S12. ^{1}H NMR spectrum of **TP-1** in DMSO-d_6 (600 MHz).

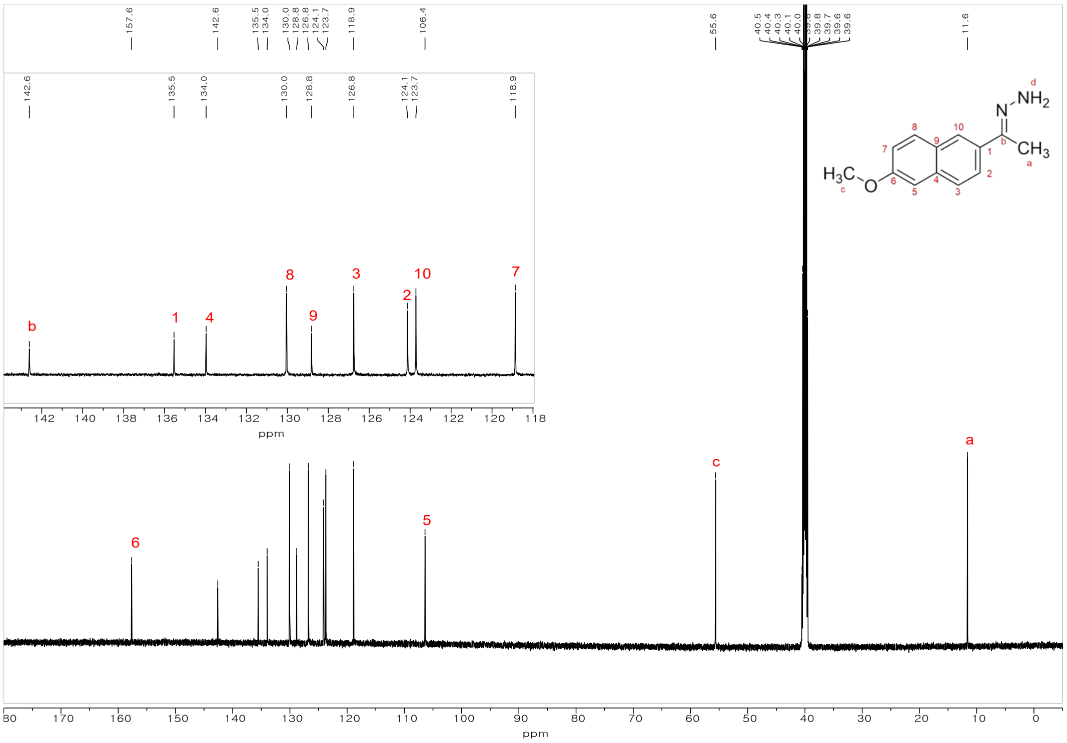

Fig. S13. ^{13}C NMR spectrum of **TP-1** in DMSO-d_6 (150 MHz).

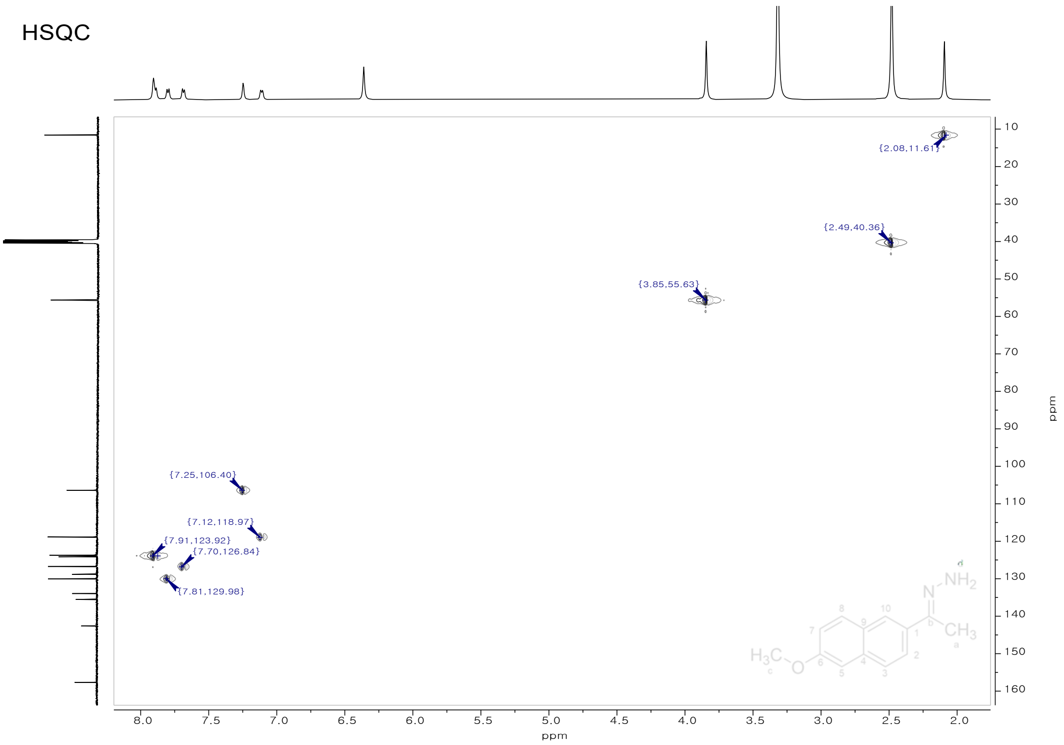

Fig. S14. HSQC NMR spectrum of **TP-1** in DMSO-d_6.

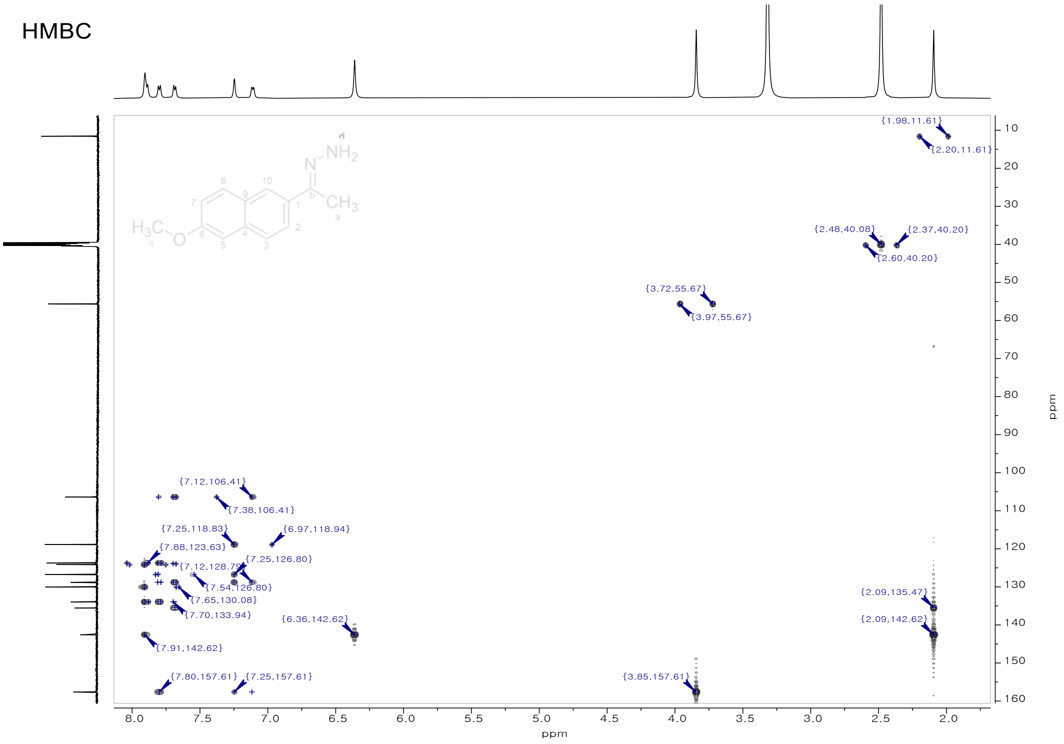

Fig. S15. HMBC NMR spectrum of **TP-1** in DMSO-d_6.

[Mass Spectrum]
Data : EI-D630 Date : 02-Aug-2023 10:27
RT : 0.55 min Scan# : (10,13)
Elements : C 100/0, H 100/0, N 5/0, O 5/0
Mass Tolerance : 10ppm, 5mmu if m/z < 500, 10mmu if m/z > 1000
Unsaturation (U.S.) : -0.5 - 20.0

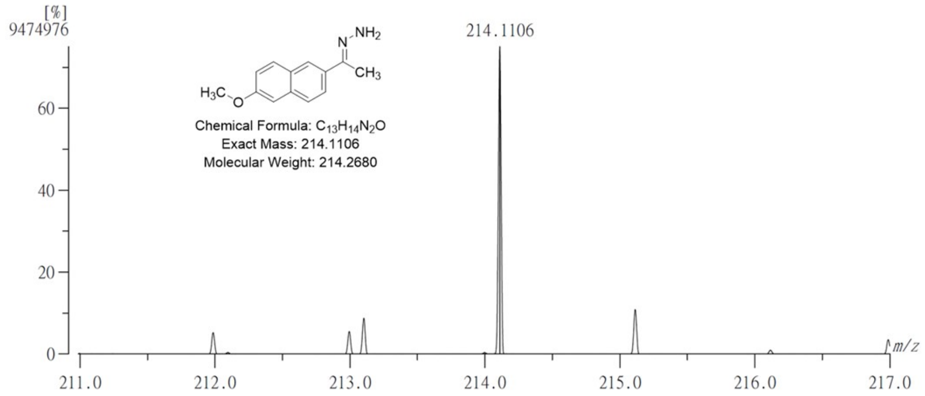

Observed m/z	Int%	Err[ppm / mmu]	U.S.	Composition
1 214.1106	75.08	+6.2 / +1.3	8.5	C11 H12 N5
2		-0.1 / -0.0	8.0	C13 H14 N2 O
3		+18.7 / +4.0	4.0	C8 H14 N4 O3
4		+12.5 / +2.7	3.5	C10 H16 N O4
5		-21.2 / -4.5	-0.5	C4 H16 N5 O5

Fig. S16. High-resolution mass spectrum of **TP-1**.

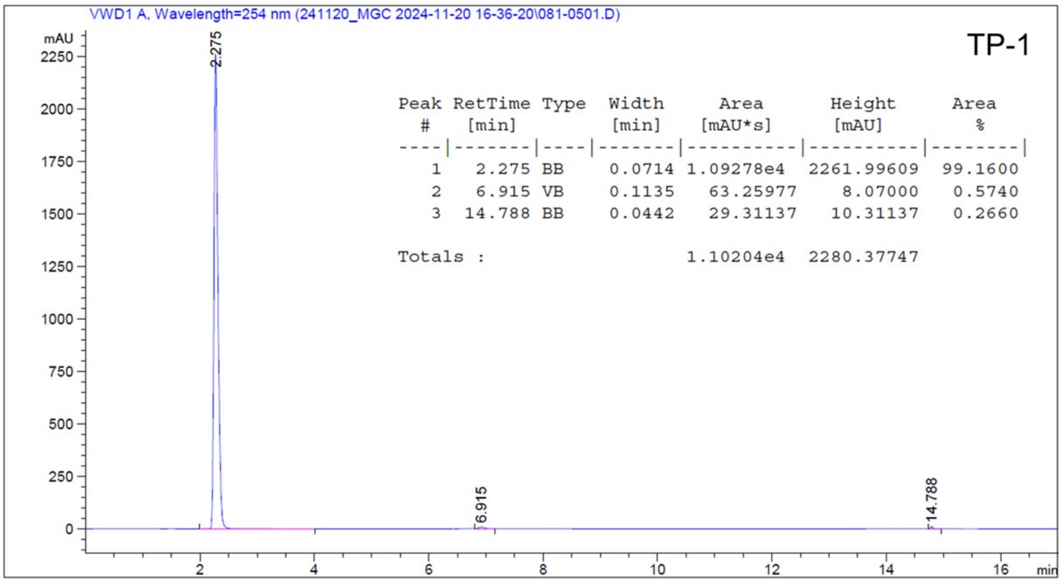

Peak #	RetTime [min]	Type	Width [min]	Area [mAU*s]	Height [mAU]	Area %
1	2.275	BB	0.0714	1.09278e4	2261.99609	99.1600
2	6.915	VB	0.1135	63.25977	8.07000	0.5740
3	14.788	BB	0.0442	29.31137	10.31137	0.2660
Totals :				1.10204e4	2280.37747	

Fig. S17. HPLC chromatogram of **TP-1**. Column: C18 column, mobile phase: H_2O:CH_3CN = 8:2 (*v/v*), flow rate: 1 mL/min, detection: Abs at 254 nm.

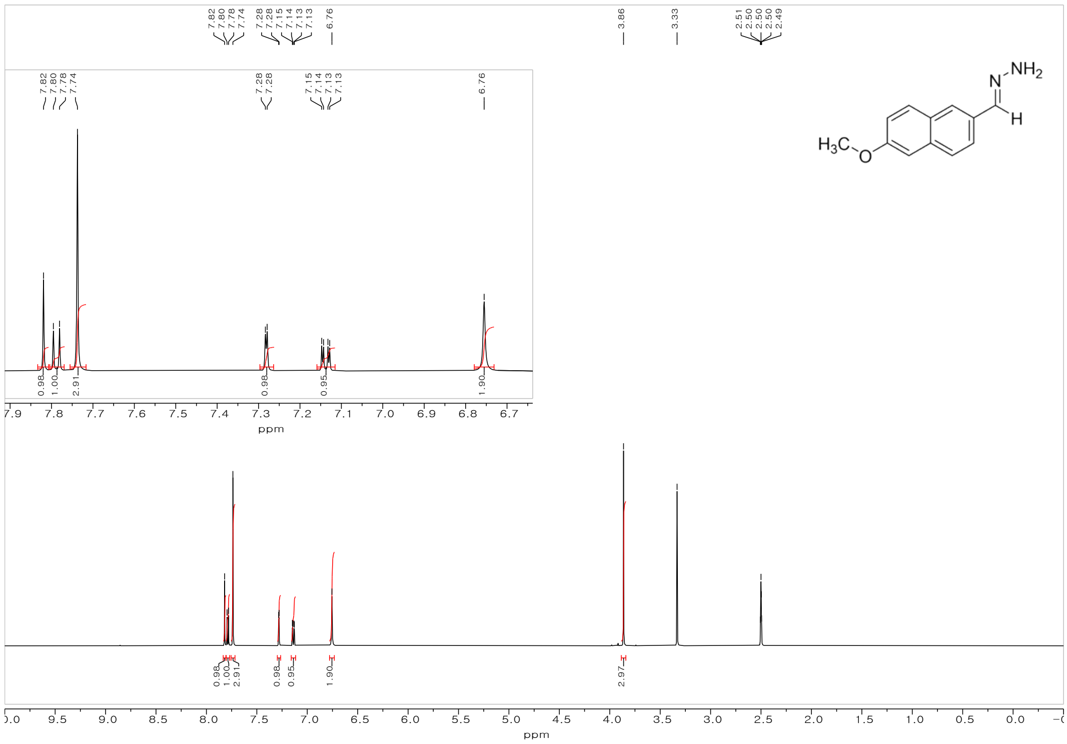

Fig. S18. ^{1}H NMR spectrum of **TP-2** in DMSO-d_6 (600 MHz).

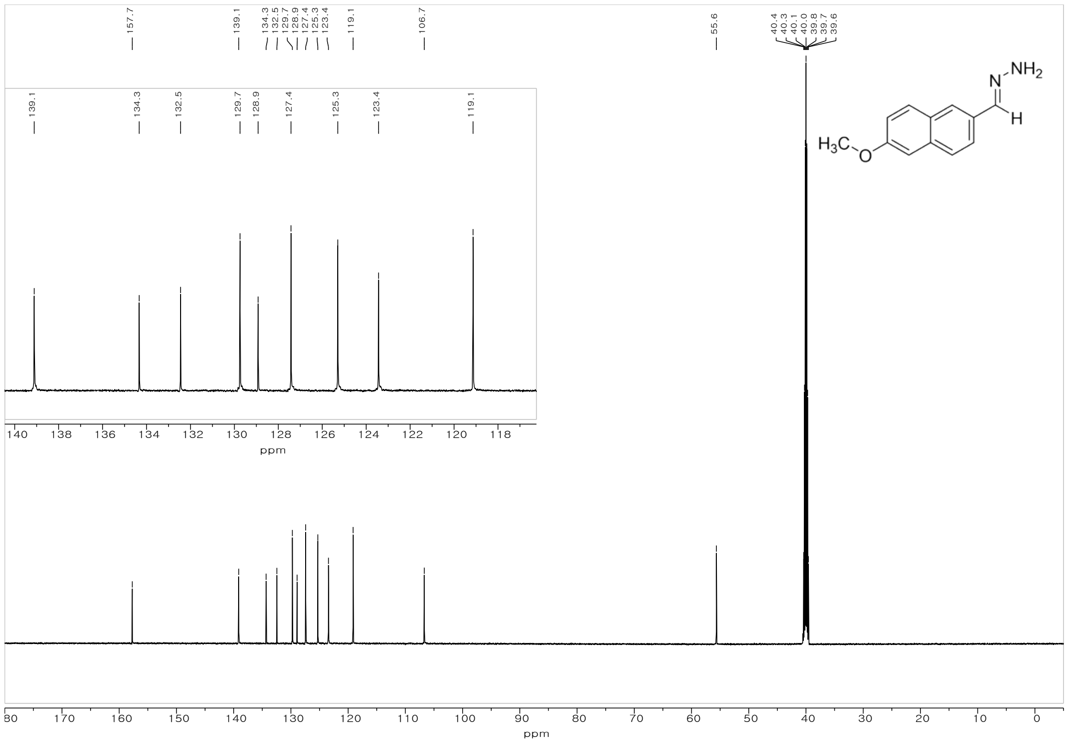

Fig. S19. ^{13}C NMR spectrum of **TP-2** in DMSO-d_6 (150 MHz).

[Mass Spectrum]
Data : EI-D629 Date : 02-Aug-2023 10:20
RT : 0.48 min Scan# : (9,19)
Elements : C 100/0, H 100/0, N 5/0, O 5/0
Mass Tolerance : 10ppm, 5mmu if m/z < 500, 10mmu if m/z > 1000
Unsaturation (U.S.) : -0.5 - 20.0

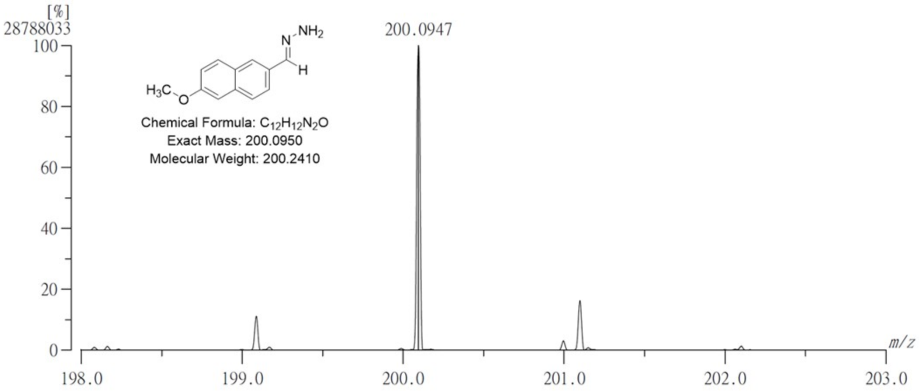

	Observed m/z	Int%	Err[ppm / mmu]	U.S.	Composition
1	200.0947	100.00	+5.4 / +1.1	8.5	C10 H10 N5
2			-1.3 / -0.3	8.0	C12 H12 N2 O
3			+18.8 / +3.8	4.0	C7 H12 N4 O3
4			+12.1 / +2.4	3.5	C9 H14 N O4
5			-24.0 / -4.8	-0.5	C3 H14 N5 O5

Fig. S20. High-resolution mass spectrum of **TP-2**.

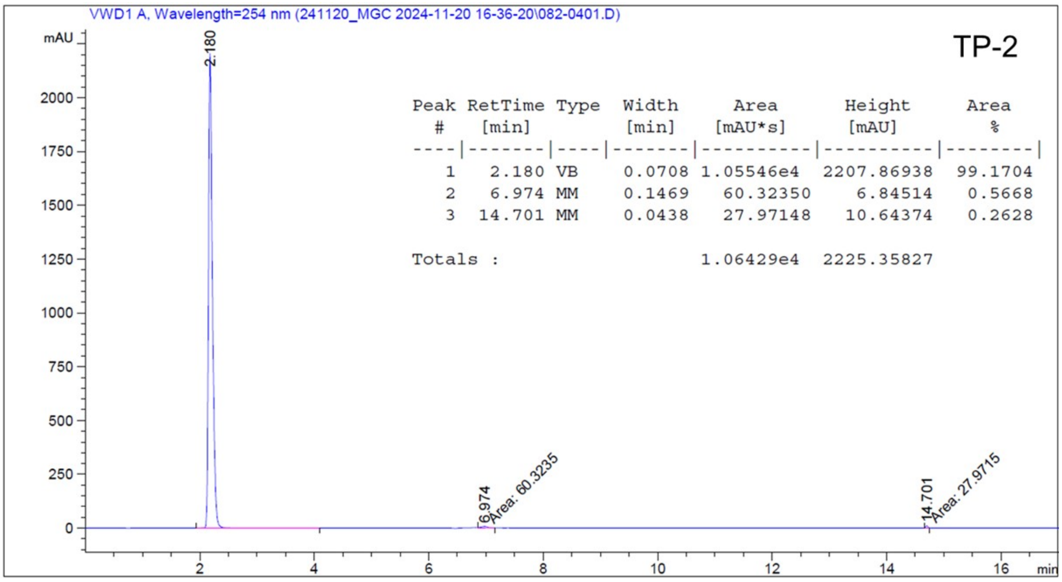

Peak #	RetTime [min]	Type	Width [min]	Area [mAU*s]	Height [mAU]	Area %
1	2.180	VB	0.0708	1.05546e4	2207.86938	99.1704
2	6.974	MM	0.1469	60.32350	6.84514	0.5668
3	14.701	MM	0.0438	27.97148	10.64374	0.2628
Totals :				1.06429e4	2225.35827	

Fig. S21. HPLC chromatogram of **TP-2**. Column: C18 column, mobile phase: H_2O:CH_3CN = 8:2 (*v/v*), flow rate: 1 mL/min, detection: Abs at 254 nm.

References

[S1] Y. K. Agrawal and V. J. Bhatt, *Analyst*, 1986, **111**, 761–765.

[S2] B. Rezaei, S. Meghdadi and N. Majidi, *Spectrochim. Acta Part A,* 2007, **67**, 92–97.

[S3] H. D. Revanasiddappa and T. N. K. Kumar, *Anal. Sci.*, 2002, **18**, 1131–1135.

[S4] S. Ge, P. Dai, J. Yu, Y. Zhu, J. Huang, C. Zhang, L. Ge and F. Wan, *Intern. J. Environ. Anal. Chem.*, 2010, **90**, 1139–1147.

[S5] P. Nagaraja, N. G. S. Al-Tayar, A. Shivakumar, A. K. Shresta and A. K. Gowda, *J. Mex. Chem. Soc.*, 2009, **53**, 201–208.

[S6] Y. J. Lee, M. G. Choi, J. H. Yoo, T. J. Park, S. Ahn and S.-K. Chang, *J. Photochem. Photobiol. A*, 2020, **394**, 112471.

[S7] J. H. Yoo, Y. J. Lee, K. M. Lee, M. G. Choi, T. J. Park and S.-K. Chang, *New J. Chem.*, 2021, **45**, 603–609.

[S8] W. H. Melhuish, *J. Phys. Chem.*, 1961, **65**, 229–235.
